# T-condylar fractures of the distal humerus in children: does early motion affect final range of motion?

**DOI:** 10.1007/s11832-014-0576-1

**Published:** 2014-03-19

**Authors:** Nicholas A. Beck, Theodore J. Ganley, Scott McKay, Lauren Tomlinson, Jaimo Ahn, John M. Flynn, Keith Baldwin

**Affiliations:** 1 Department of Orthopaedic Surgery, University of Minnesota, 2450 Riverside Avenue South, Suite R200, Minneapolis, MN 55454 USA; 2 Division of Orthopaedic Surgery, Texas Children’s Hospital, Houston, TX, USA; 3 Department of Orthopaedic Surgery, Hospital of the University of Pennsylvania, 3400 Spruce Street, 2 Silverstein, Philadelphia, PA 19104 USA; 4 Division of Orthopaedic Surgery, The Children’s Hospital of Philadelphia, 34th and Civic Center Blvd., Philadelphia, PA 19104 USA; 5 Department of Orthopaedic Surgery, Baylor College of Medicine, Texas Children’s Hospital, 6701 Fannin St. CCC650.01, Houston TX, 77030 USA

**Keywords:** T-condylar, T-condylar distal humerus fractures, Upper extremity fractures

## Abstract

**Purpose:**

T-condylar fractures of the distal humerus are infrequent injuries in children. There are little data regarding outcomes in this age group. The adult literature demonstrates a high rate of postinjury stiffness. We describe a large series of T-condylar fractures in children and set out to identify factors that influence the postoperative range of motion (ROM) in children. Our hypothesis was that starting motion early (<3 weeks) would favorably influence the postoperative ROM.

**Methods:**

Patients were identified based on the Current Procedural Terminology (CPT) code for ORIF of supracondylar distal humerus fractures with intracondylar extension (24546). Patient records and radiographs were reviewed to determine the demographics, fracture characteristics, surgical approach and fixation, and postoperative immobilization time. Our outcome measure was ROM in flexion/extension at 3 months, 6 months, 1 year, and final follow-up. Patients were analyzed by Morrey’s criteria of −30° extension and 130° flexion to assess for postoperative elbow stiffness.

**Results:**

Thirty-eight potential patients from 1992 to 2010 were identified with specific T-condylar patterns. Twelve patients were excluded due to insufficient follow-up or lack of final ROM data. Our cohort included 26 patients (average age 13.4 years). The average postoperative immobilization time was 3.4 weeks (range 0.9−12 weeks). At the final follow-up, patients had −12° average extension and 130° average flexion. Nine patients (35 %) were stiff and 17 patients (65 %) had functional motion postoperatively. At 3 and 6 months, starting motion early yielded better flexion and extension ROM. Late-motion patients obtained similar results at the 1-year follow-up. Open fractures, gender, and age were all not significantly associated with elbow stiffness in our series, given the limited numbers.

**Conclusion:**

Early ROM was associated with an earlier gain of functional motion without clear adverse consequences. Despite similar findings at the final follow-up, practitioners should consider instituting early ROM protocols to decrease the duration of stiffness and potential disability for the child and the family.

## Introduction

T-condylar fractures of the humerus are rare injuries in children and adolescents. A review of 300 consecutive cases of fractures of the elbow in children showed that this injury pattern occurs in only 2 % of cases [1]. The mechanism of injury has been described as being similar to that for supracondylar fractures but from a higher energy injury [2–4]. This fracture pattern is difficult to treat in both children and adults because of its intra-articular extension. The adult literature shows that these fractures often result in postinjury elbow stiffness [2–4]. Additionally, these fractures can be difficult to diagnose in children younger than 8 years of age, as the ossification centers are cartilaginous and not visible on routine radiographs [5–7].

Historically, operative treatment was discouraged because of poor outcomes [8]. However, internal fixation techniques and implants have advanced such that these fractures can be successfully treated surgically [9]. For instance, studies have shown that screw fixation and plating have better outcomes than wire fixation [10]. Various approaches have been utilized for open reduction of these fractures. Posteromedial triceps slide (Bryan–Morrey type) and olecranon osteotomy approaches have resulted in better postoperative extension range of motion (ROM) than the triceps splitting approach in one study of T-condylar fractures [11]. However, a recent review found no difference in the final ROM between the Bryan–Morrey approach, olecranon osteotomy, triceps splitting, paratricipital, and triceps-reflecting anconeus pedicle (TRAP) when pooling the available literature on all types of intra-articular distal humerus fractures [12]. Factors shown to negatively affect outcome include open fractures [5], comminution, polytrauma, and associated injury to the ipsilateral arm [8].

We describe a large series of patients at a tertiary care institution and set out to identify factors that influence the postoperative ROM in children. Our hypothesis was that starting motion early (<3 weeks) would favorably influence the postoperative ROM.

## Methods and materials

After Institutional Review Board approval, we performed a retrospective review of all pediatric patients aged 0–18 years treated surgically at our pediatric trauma center between 1/1/1992 and 5/1/2010. We identified patients via a query of our outpatient billing database for the Current Procedural Terminology (CPT) code 24546 (ORIF of supracondylar distal humerus fractures with intracondylar extension). Patients were included if they received surgical treatment for a T-condylar fracture with a completed operative note at our institution and had at least 2 months of clinical follow-up. Surgeries were performed by six attending pediatric orthopedic surgeons. Full details of the patient characteristics and outcome are highlighted in Table 1.

**Table 1:** Patient characteristics

Pt. number	Age (years)	Gender	MOI	Approach/fixation	Time to ROM (weeks)	Follow-up (months)	Open fracture	Final ROM (°)	Complications
1	8.5	F	FOOSH^a^	BM^b^ screws/wires	7.2	16.6	N	5–140	None
2	13.3	M	Football	OO^c^ plate/screw	1.4	5.6	N	5–150	None
3	15.2	M	Hockey	TS± plates/screw	1.6	2.1	N	20–95	Stiffness
4	12.2	M	FOOSH^a^	OO^c^ plate/screw	1.3	14.0	N	25–130	Wound infection olecranon osteotomy non-union
5	14.8	M	FOOSH^a^ (skateboarding)	TS± plate/screw	1.6	9.8	Y	35–100	Heterotopic bone formation, stiffness, MUA, ulnar neurapraxia
6	12.4	M	Fall off bike	OO^c^ plate/screw	2.7	18.1	N	3–150	None
7	15.0	M	Fall off trampoline	OO^c^ plate/screw	4.8	10.1	N	5–130	Symptomatic hardware
8	12.9	F	Fall from go-kart	OO^c^ plate/screw	3.8	11.5	N	15–120	Stiffness
9	12.2	M	Fall from bike	OO^c^ Plate/screw	2.3	9.3	Y	2–130	None
10	14.1	F	Fall (gymnastics)	CRPP±± revised to screws with TS±	4.2	5.8	N	0–115	Stiffness, required LOA/MUA
11	14.0	M	FOOSH^a^	OO^c^ plate/screw	1.4	16.1	N	10–130	Symptomatic hardware
12	10.8	M	FOOSH^a^	BM^b^ plate/screw	2.3	3.0	N	30–130	None
13	13.4	M	Fall during skateboarding	OO^c^ plate/screw	10.1	8.7	N	20–120	Symptomatic hardware, stiffness
14	14.0	M	Fell off a bike	OO^c^ plate/screw	1.9	5.0	N	10–150	Refracture of olecranon osteotomy
15	12.8	M	ATV injury	BM^b^ plate/screw	1.3	10.3	N	10–100	Symptomatic hardware, stiffness
16	12.6	M	FOOSH^a^ (ice)	BM^b^ plate/screw	2.9	12.1	N	5–140	None
17	13.1	M	Fall during sledding	TS± screws only	1.3	17.0	N	5–140	None
18	14.3	M	Fall (roller hockey)	TS± plate/screw	2.3	11.5	Y	5–145	None
19	14.8	M	Fall off bike	OO^c^ plate/screw	0.9	5.5	N	5–150	None
20	14.1	M	Fall (skateboarding)	OO^c^ plate/screw	2.9	7.2	N	15–150	None
21	14.9	M	ATV injury	BM^b^ plate/screw	2.0	13.8	Y	20–150	None
22	13.5	M	Fall off bike	TS± plate/screw	3.5	5.8	N	20–120	Ulnar claw hand, osteomyelitis, stiffness
23	13.2	M	FOOSH^a^	TS± plate/screw	12.3	2.8	Y	25–90	Stiffness
24	16.4	F	Fall from height	OO^c^ plate/screw	4.9	6.4	N	0–120	Wound dehiscence, stiffness
25	13.3	M	FOOSH^a^	OO^c^ plate/screw	3.0	2.8	Y	15–145	None
26	12.5	F	FOOSH^a^	Medial screws/wires	5.5	27.6	Y	10–150	None

± Triceps slide; ±± closed reduction percutaneous pinning

^a^Fall on outstretched hand

^b^Morrey slide exposure

^c^Olecranon osteotomy

From the patients’ medical records, we recorded the following demographics: age, gender, mechanism of injury, dominant or non-dominant arm, and associated injuries. Operative notes were reviewed to determine: AO fracture classification [11], open fractures, time from injury to surgery, surgical approach, articular congruity, type of surgical fixation, ulnar nerve transposition, intraoperative ROM, surgical complications, and postoperative immobilization. Patients’ outpatient charts and radiographs were reviewed to determine: quality of reduction, length of immobilization, time to motion, ROM (flexion, extension, supination, and pronation) at each follow-up, and complications, such as loss of reduction, heterotopic ossification, arthrofibrosis, and abnormal growth of the trochlea.

Heterotopic ossification was not classified in the radiographic review because it was not a prominent feature of the postoperative radiographs. The quality of reduction was classified as flexed or extended based on the anterior humeral line passing posterior or anterior to the capitellum, respectively.

ROM in flexion and extension was recorded for each patient at 3 months, 6 months, 1 year, and final follow-up. Patients were analyzed by Morrey’s criteria of −30° extension and 130° flexion to assess for postoperative elbow stiffness [13]. At the final follow-up, patients were grouped into two cohorts for comparison: good motion cohort (>−30° of extension and >130° of flexion) and stiff cohort (flexion of <130° and/or extension of <−30°). Short immobilization was defined as starting motion prior to 3 weeks postoperatively and extended immobilization was defined as starting motion after 3 weeks postoperatively.

Analysis was performed comparing the stiff cohort to the good motion cohort. In addition, ROM at 3 months, 6 months, and 12 months was compared between patients with short immobilization and those with extended immobilization.

### Statistical analysis

Demographic characteristics were summarized by standard descriptive summaries (e.g., means and standard deviations for continuous variables such as age and percentages for categorical variables such as gender). For variables in which the outcome of interest is binary or categorical, a Chi0square test was used with Yates’ correction. Fisher’s exact test was used when there were cell values of <5. Continuous normally distributed variables were measured with *t*-tests for independent samples in cases where there are only two groups and one-way analysis of variance (ANOVA) in cases where there are more than two groups. Statistical significance was set at an alpha level of *p* = 0.05. All statistics were calculated with SPSS version 18 (SPSS Inc., Chicago, IL).

## Results

Between January 1992 and May 2010, we identified 38 potential patients within the appropriate age range treated at our institution with a fracture of the distal humerus in specific T-condylar patterns. Twelve patients were excluded due to insufficient follow-up or lack of ROM data. Twenty-six patients were included into our study, with a mean age of 13.4 years (range 8.5–16.4 years). The average follow-up was 9.94 months (range 2.8–27.6 months). There were 7 (27 %) open fractures. Six fractures were classified as OTA/AO 13-C1 and 18 were classified as OTA/AO 13-C2. Two fractures were not classified.

Details of the fixation and approach are shown in Table 1.

After applying Morrey’s criteria to our patients’ ROM data at the final follow-up, nine patients (35 %) were stiff. The remaining 17 patients (65 %) had functional ROM. A detailed breakdown of these groups is shown in Table 2.

**Table 2:** Patients with stiffness (<30° extension or <130° flexion or both) versus those patients with functional range of motion (ROM) at final follow-up

	Stiff patients (*n* = 9)	Good motion (*n* = 17)	*p*-Value
Age	13.6 years	13.2 years	0.721
Gender	2/9 female	4/17 female	0.999
Side	6/9 left	8/17 left	0.429
Open fracture	2/9 open	5/17 open	0.999
Final extension	16.7°	10°	0.091
Final flexion	111.1°	141.2°	<0.001
Final arc	94°	123.5°	0.033
Time to motion	4.5 weeks	2.8 weeks	0.138
Total follow-up	7.6 months	11.2 months	0.171

**Fig. 1:**
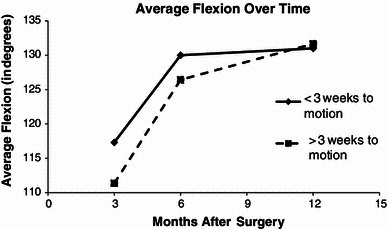
Average flexion at 3, 6, and 12 months

**Fig. 2:**
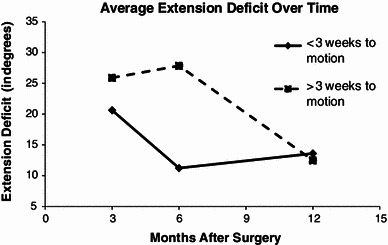
Average extension deficit at 3, 6, and 12 months

Of the 26 patients with postoperative immobilization data, 16 patients had a short immobilization (<3 weeks) and ten patients had an extended immobilization (longer than 3 weeks). The average time to motion in the short immobilization group was 1.9 weeks and the average time to motion in the extended immobilization group was 5.9 weeks. Figures 1 and 2 show the average flexion and extension, respectively, at 3 months, 6 months, and 12 months postoperatively. For both flexion and extension, the short immobilization group had better motion at 3 and 6 months postoperatively. However, at 12 months, the extended immobilization group’s ROM caught up. Both groups ended up with similar flexion of ~131° and similar extension of ~13° at 12 months. In the shorter immobilization group, a quarter of patients ended up stiff, as opposed to half of the patients in the longer immobilization group. The overall arc of motion at the final follow-up was 120.9° in the short immobilization group versus 101.5 in the longer immobilization group (*p*-value 0.156). These findings may be explained by the fact that 40 % of the longer immobilization time patients had limited follow-up (<6 months) compared to only 25 % of the shorter immobilization time patients. Other factors investigated were not significant predictors of stiffness.

## Discussion

T-condylar fractures are a rare injury in children. Most of them occur in the young to mid adolescents. As such, this large series from a tertiary care center is relevant. The relative rarity of the injury with the poor outcomes relative to other children’s fractures may warrant a registry in order to answer questions more rigorously on how to improve the outcomes of these difficult fractures. Because loss of motion is thought to be the greatest complication from these injuries, we sought to find factors that influenced the postoperative ROM.

First, we found that immobilization <3 weeks had a favorable association with motion at the 3-month and 6-month time periods, but this effect was not significant at 1 year. Early motion after fixation is advocated by many authors as a standard of care in adult distal humerus fractures because it affects the final ROM [14–16]. Interestingly, in our pediatric and adolescent population, the duration of immobilization did not seem to affect ROM at the 1-year follow-up. Adult series show fractures with more articular comminution, whereas the articular comminution in our patient population is less substantial. The simplicity of the fracture pattern and better bone quality may enable pediatric orthopedists to expect superior outcomes compared to adult patients. However, these fractures remain an enigma because of their vexing propensity towards stiffness seldom encountered in other pediatric injuries.

The small size and heterogeneity of our population created difficulty with inferential statistics. We noted that two-thirds of the patients who became stiff had left-sided injuries, compared to only 8/17 patients in the good motion cohort (less than half). Though our study was too small to detect this difference, we have noted anecdotally that our population of children in general obtains the majority of their motion through normal play and not structured physical therapy. Hence, sidedness may play a role in functional recovery from this injury that was not fully appreciated in this current series. This was also seen in the next largest published series of these fractures [11].

Our study was too small to investigate other factors, such as surgical approach and reduction quality. We had one patient who had an olecranon osteotomy non-union and one who refractured. This complication, though well recognized in adults, is less commonly seen in children. However, because the osteotomy provides the potential for additional complications, and little advantage in pediatric patients, our institution has moved away from performing an osteotomy. We now prefer a Morrey slide technique, as it eliminates the possibility of a non-union and provides acceptable exposure in the T-condylar humerus fracture in which the articular block is not as comminuted [17]. We have demonstrated that early motion effects early outcome in ROM. Though our findings are limited by the study design, we recommend, where possible, fixation rigid enough to allow early motion.

## Conclusion

Range of motion (ROM) initiated within 3 weeks of open reduction of T-condylar fractures in children and adolescents was associated with an earlier gain of functional motion compared with those who began motion later than 3 weeks. However, at 1 year, the late motion and early motion groups were equivalent. This uncommon injury continues to be vexing to pediatric orthopedic surgeons due to its propensity for stiffness and complications. A large multicenter registry study may be valuable to discern the optimal care of these patients, as the injury is rare and produces suboptimal outcomes when compared to other injuries of childhood and adolescence.
